# Supplementary material for: CovDif, a Tool to Visualize the Conservation between SARS-CoV-2 Genomes and Variants
Source: Viruses. 2022 Mar 9;14(3):561. doi: 10.3390/v14030561 (PMC8955889; doi:10.3390/v14030561)
Supplement: Supplementary file 1 [file viruses-14-00561-s001.zip › Supplemental_Information_v2.0.pdf]

CovDif, a tool to visualize the conservation between SARS-CoV-2 genomes and variants

Luis F. Cedeño-Pérez[1], Laura Gómez-Romero[2]

Clade	Number of genomes	Selected dates of submission
G	9427	Between 2020-12-01 and 2021-01-05
GH	9823	Between 2020-12-12 and 2021-01-05
GR	9982	Between 2020-12-10 and 2020-12-14
GV	9555	Between 2020-12-23 and 2020-01-05
L	3378	All genomes available until 2020-01-05
O	3532	All genomes available until 2020-01-05
S	6059	All genomes available until 2020-01-05
V	3940	All genomes available until 2020-01-05

Supplementary Table S1. Number of genomes downloaded per each clade. The dates of submission were restricted when more than 10,000 genomes per clade were available. The selected submission dates and the number of genomes per clade are shown

Variant	Number of genomes	Selected dates of submission
b.1.1.7	9889	Between 2021-03-03 and 2021-03-05
b.1.351	2866	All genomes available until 2021-03-08
p.1	616	All genomes available until 2021-03-08
b.1.525	418	All genomes available until 2021-03-08
b.1.427	9971	All genomes available until 2021-03-08
b.1.1.529	8558	Between 2022-02-16 and 2022-02-18

Supplementary Table S2. Number of genomes downloaded per each variant. The dates of submission were restricted when more than 10,000 genomes per variant were available. The selected submission dates and the number of genomes per variant are shown.

Kit	Oligo name	Strand	Sequence (5'-3')	Position
CDC	2019-nCoV_N1	F	GACCCCAAAATCAGCGAAAT	28287-28306
		R	TCTGGTTACTGCCAGTTGAATCTG	28335-28358
	2019-nCoV_N2	F	TTACAAACATTGGCCGCAAA	29164-29183
		R	GCGCGACATTCCGAAGAA	29213-29230
	2019-nCoV_N3	F	GGGAGCCTTGAATACACCAAAA	28681-28702
		R	TGTAGCACGATTGCAGCATTG	28732-28752
LKS	HKU	F	TAATCAGACAAGGAACTGATTA	29145-29166
		R	CGAAGGTGTGACTTCCATG	29236-29254
Biobasic	E Sarbeco	F	ACAGGTACGTTAATAGTTAATAGCGT	26269-26294
		R	ATATTGCAGCAGTACGCACACA	26360-26382
Reza Mollaei H. *et al* 2020	Orf1ab	F	CTAGGACCTCTTTCTGCTCA	10802-10821
		R	ACACTCTCCTAGCACCATCA	11371-11391
	S	F	CCCTGTTGCTATTCATGCAG	23422-23441
		R	CCCTATTAAACAGCCTGCAC	23499-23518
	E	F	GGAAGAGACAGGTACGTTAA	26262-26281
		R	AAGGTTTTACAAGACTCACG	26388-26407
	N	F	CCTCTTCTCGTTCCTCATCA	28818-28837
		R	CCTGGTCCCCAAAATTTCCT	29122-29141
	RdRp	F	CATCTCACTTGCTGGTTCCT	4777-4796
		R	CATCTCACTTGCTGGTTCCT	4951-4973
Roujian Lu. *et al* 2020	Orf1ab	F	AGAAGATTGGTTAGATGATGATAGT	3193-3217
		R	TTCCATCTCTAATTGAGGTTGAACC	3286-3310
China CDC	N	F	GGGGAACTTCTCCTGCTAGAAT	28881-28902
		R	CAGACATTTTGCTCTCAAGCTG	28958-28979
	ORF1ab	F	CCCTGTGGGTTTTACACTTAA	13342-13362
		R	ACGATTGTGCATCAGCTGA	13442-13460
Japan-NIID	2019-nCOV_N	F	AAATTTTGGGGACCAGGAAC	29125-29144
		R	TGGCAGCTGTGTAGGTCAAC	29263-29282
Thailand-WH	NIC_N	F	CGTTTGGTGGACCCTCAGAT	28320-28339
		R	CCCCACTGCGTTCTCCATT	28358-28376

Supplementary Table S3. RT-qPCR primers analyzed in this study. For each primer pair either the commercial kit names or the academic reference is mentioned, along with its name, orientation, sequence and targeted genomic region

Start position	End position	Median frequency value
293	313	0.847
425	445	0.825
3017	3037	0.294
6266	6286	0.822
8762	8782	0.889
11063	11083	0.865
14388	14408	0.291
14785	14805	0.889
20248	20268	0.897
21235	21255	0.824
22207	22227	0.824
23383	23403	0.290
25543	25563	0.810
26781	26801	0.823
28822	28899	0.878
28912	28932	0.825
29625	29645	0.822

Supplementary Table S4. Drop regions with values lower than 0.9 at the conservation landscape of SARS-CoV-2 population genomes. The start and end positions are shown for each drop region. The median value is obtained as the median frequency value for all kmers immersed in each region.

Clade	Mutation name	Reference kmers start positions	Median frequency at conservation landscape	Median frequency at differential landscape	Already reported?
S, L	C8782T	8763-8782	S: 0.001<br>L: 0.998	0.996	Yes
S	T28144C	28125-28144	S: 0.001	0.998	Yes
L, G, GH, GR, GV	C241	222-241	G: 0.001<br>GH: 0.004<br>GR, GV: 0<br>L: 0.998	0.997	Yes
	C3037	3018-3037	G, GH, GR, GV: 0<br>L: 0.998	0.999	Yes
	A23403	23384-23403	G, GH, GR, GV: 0<br>L: 0.999	0.999	Yes
L, V	G11083	11064-11083	L: 0.999<br>V: 0	0.999	Yes
	G26144	26125-26144	L: 0.998<br>V: 0	0.999	Yes
L	T28144	28125-28144	L: 0.999	0.998	Yes
V	G11083T	11064-11083	V: 0	0.999	Yes
	NSP6-L37F	11064-11083	V: 0	0.999	Yes
	NS3-G251V	26125-26144	V: 0	0.999	Yes
G, GH, GR, GV	S-D614G	23384-23403	G, GH, GR: 0	0.999	Yes
GH	G25563T	25544-25563	GH: 0	0.998	Yes
	NS3-Q57H	25544-25563	GH: 0	0.998	Yes
GR	G28882A	28862-28883	GR: 0	0.998	Yes
	N-G204R	28862-28883	GR: 0	0.998	Yes
GV	C22227T	22208-22227	GV: 0	0.999	Yes
	S-A222V	22208-22227	GV: 0	0.999	Yes
G, GH, GR, GV**	NA	14389-14408	G: 0.001***<br>GH, GR, GV: 0***	0.999	No
GR**	NA	294-313	GR: 0.167	0.831	No
GV**	NA	185-204	GV: 0.374	0.623	No

GV**	NA	426-445	GV: 0.003***	0.996	No
GV**	NA	6267-6286	GV: 0.001***	0.998	No
GV**	NA	21236-21255	GV: 0.002***	0.996	No
GV**	NA	26782-26801	GV: 0.001***	0.998	No
GV**	NA	27925-27944	GV: 0.305	0.692	No
GV**	NA	28913-28932	GV: 0.002***	0.997	No
GV**	NA	29626-29645	GV: 0.001***	0.994	No
V**	NA	14786-14805	V: 0.042	0.955	No

** Mutations not-annotated as clade-associated mutations

*** Mutations not-annotated as clade-associated mutations and presenting a frequency at the conservation landscape lower than 0.01

Supplementary Table S5. Frequency of reference kmers across clade-specific conservation landscapes. If a mutation is observed in more than one clade, the frequency values observed in the corresponding conservation landscapes are listed in the same order as the clades.

Variant	Mutation name	Median frequency at conservation landscape	Median frequency at differential landscape
B.1.525	orf1ab-L4715F	0.011	0.228
	S-Q52R	0.310***	0.687
	S-Q677H	0.013	0.984
	S-F888L	0.017	0.981
	E-L21F	0.040	0.959
	E-I82T	0.993***	0.006
	del-28278:3	0.037	0.985
B.1.351	S-D80A	0.009	0.988
	S-D215G	0.059	0.939
	S-K417N	0.039	0.980
	S-A701V	0.008	0.991
	E-P71L	0.010	0.987
	ORF1a-K1655N	0.023	0.975
B.1.427/B.1.429	ORF1a-T265I	0.002	0.995
	ORF1a-S3158T	0.721***	0.278
	ORF1a-I4205V	0.281***	0.717
	ORF1b-P976L	0.721***	0.278
	ORF1b-P314L	0.232***	0.228
	ORF1b-D1183Y	0.010	0.989
	ORF3a-Q57H	0.006	0.993
	S-D614G	0.0003	0.033
	S-L452R	0.021	0.978
	S-S13I	0.023	0.976
	S-W152C	0.056	0.942
B.1.1.7	ORF1ab-T1001I	0.012	0.986
	ORF1ab-A1708D	0.007	0.987
	ORF1ab-I2230T	0.039	0.960
	del:21991:3	0.065	0.930
	S-A570D	0.010	0.989
	S-P681H	0.010	0.987
	S-T716I	0.010	0.989
	S-S982A	0.008	0.991
	S-D1118H	0.006	0.993
	Orf8-Q27stop	0.024	0.973
	Orf8-R52I	0.025	0.971
	Orf8-Y73C	0.006	0.99
	N-D3L	0.022	0.983

	N-S235F	0.015	0.982
P.1	ORF1ab-S1188L	0.024	0.973
	ORF1ab-K1795Q	0.006	0.993
	S-T20N	0.008	0.987
	S-P26S	0.016	0.978
	S-D138Y	0.016	0.980
	S-R190S	0.077	0.921
	S-K417T	0.019	0.980
	S-H655Y	0.004	0.992
	S-T1027I	0.009	0.989
	S-L18F	0.008	0.987
	N-P80R	0.017	0.982
	ORF3a-G174C	1***	0.913
	ORF8-E92K	0.016	0.983
B.1.1.529	del:6513:3	0.73	0.266
	del:11283:9	0.23	0.978
	nuc:C241T	0.02	0.018
	nuc:C3037T	0.004	0.014
	nuc:T5386G	0.69	0.987
	nuc:T13195C	0.70	0.297
	nuc:C15240T	0.99	0.002
	nuc:C25000T	0.12	0.878
	nuc:A27259C	0.0008	0.998
	nuc:C27807T	0.0004	0.998
	ORF1A:K856R	0.70	0.299
	ORF1A:A2710T	0.70	0.297
	ORF1A:T3255I	0.0005	0.999
	ORF1A:P3395H	0.008	0.991
	ORF1A:I3758V	0.69	0.302
	S:A67V	0.705	0.973
	S:T95I	0.72	0.269
	S:G339D	0.0007	0.999
	S:S371L	0.01	0.988
	S:S373P	0.009	0.989
	S:K417N	0.01	0.983
	S:N440K	0.14	0.857
	S:G446S	0.70	0.290
	S:S477N	0.02	0.973
	S:T478K	0.02	0.973
	S:E484A	0.02	0.984
	S:Q493R	0.02	0.976
	S:G496S	0.02	0.976
	S:Q498R	0.02	0.985
	S:T547K	0.70	0.299

	S:D614G	0.0002	0.033
	S:H655Y	0.0001	0.997
	S:N679K	0.0001	0.997
	S:P681H	0.0001	0.996
	S:N764K	0.007	0.992
	S:D796Y	0.002	0.997
	S:N856K	0.70	0.299
	S:Q954H	0.001	0.998
	S:N969K	0.001	0.997
	E:T9I	0.001	0.998
	M:D3G	0.71	0.281
	M:Q19E	0.27	0.726
	M:A63T	0.001	0.998
	N:RG203KR	0.0004	0.027
B.1.525, B.1.351, P.1	S-E484K	0.013, 0.078, 0.108***	0.984
B.1.351, B.1.1.7, P.1, B.1.1.529	S-N501Y	0.083, 0.012, 0.110***, 0.02	0.985
B.1.1.7, P.1, B.1.525	del:11288:9	0.023, 0.021, 0.040	0.978
B.1.351, B.1.427/B.1.429	N-T205I	0.027, 0.009	0.018
B.1.525, B.1.1.7	del:21765:6	0.024, 0.050	0.973

Supplementary Table S6. Frequency of reference kmers across lineage-specific conservation landscapes. If a mutation is observed in more than one clade, the frequency value observed in the corresponding conservation landscape is listed in the same order as the lineages.

Detection protocol	Oligo name	Strand	Genomic position	Variant with low frequency at conservation landscape
CDC	2019-nCoV_N1	F	28287-28306	NA
		R	28335-28358	NA
	2019-nCoV_N2	F	29164-29183	NA
		R	29213-29230	NA
	2019-nCoV_N3	F	28681-28702	b.1.525 (1%)
		R	28732-28752	NA
LKS		F	29145-29166	NA
		R	29236-29254	NA
Biobasic		F	26269-26294	NA
		R	26360-26382	NA
Reza Mollaei H. et al 2020	Orf1ab	F	10802-10821	NA
		R	11371-11391	NA
	S	F	23422-23441	NA
		R	23499-23518	NA
	E	F	26262-26281	b.1.1.529 (0%)
		R	26388-26407	NA
	N	F	28818-28837	NA
		R	29122-29141	NA
	RdRp	F	4777-4796	NA
		R	4951-4973	NA
Roujian Lu et al 2020	Orf1ab	F	3193-3217	NA
		R	3286-3310	NA
China CDC	N	F	28881-28902	b.1.1.7 (2%)<br>b.1.351 (2%)<br>b.1.427 (0%)<br>b.1.525 (2%)<br>p.1 (2%)<br>b.1.1.529 (0%)
		R	28958-28979	b.1.1.7 (1%)<br>b.1.427 (76%)
	Orf1ab	F	13342-13362	NA
		R	13442-13460	NA
Japan-NIID	2019-nCOV_N	F	29125-29144	NA
		R	29263-29282	NA
Thailand-WH	NIC_N	F	28320-28339	NA
		R	28358-28376	b.1.1.529 (6%)

Supplementary Table S7. Frequency of reference kmers across lineage-specific conservation landscapes for primer regions. Primers were obtained from current SARS-CoV-2 detection protocols.

SUPPLEMENTARY FIGURES

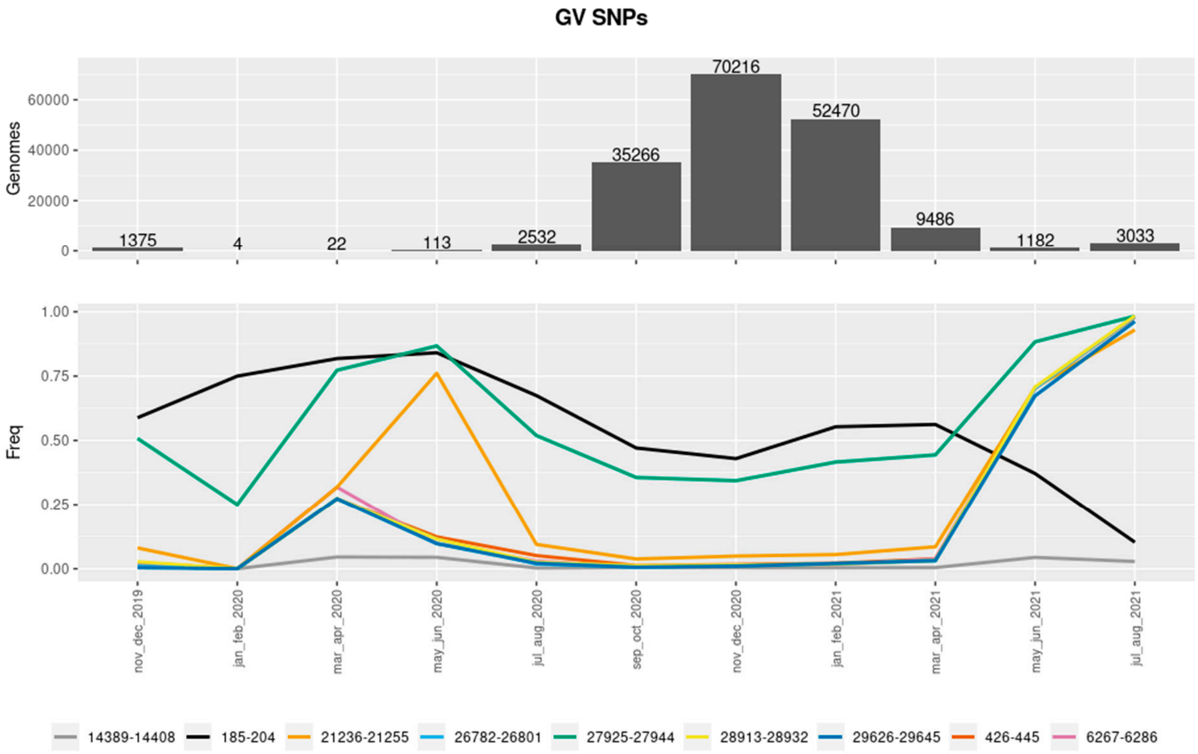

Supplementary Figure S1. Conservation landscape frequency over time for mutations of interest. The frequency at the conservation landscape (the frequency of the reference allele) over time is shown for several mutations of interest. Top track: the number of GV genomes at each time interval. Bottom track: the frequency at the conservation landscape for each mutation, each colored line represent a different mutation.
